# Daytime Sleepiness in Patients Diagnosed with Sarcoidosis Compared with the General Population

**DOI:** 10.1155/2018/6853948

**Published:** 2018-07-10

**Authors:** Andreas Hinz, Kristina Geue, Markus Zenger, Hubert Wirtz, Andrea Bosse-Henck

**Affiliations:** ^1^Department of Medical Psychology and Medical Sociology, University of Leipzig, Leipzig, Germany; ^2^Faculty of Applied Human Studies, University of Applied Sciences Magdeburg and Stendal, Stendal, Germany; ^3^Integrated Research and Treatment Center (IFB) Adiposity Diseases-Behavioral Medicine, Medical Psychology and Medical Sociology, University of Leipzig, Leipzig, Germany; ^4^Department of Respiratory Medicine, University of Leipzig, Leipzig, Germany

## Abstract

**Background:**

The aim of this study was to analyze daytime sleepiness in a sample of patients diagnosed with sarcoidosis.

**Methods:**

A sample of 1197 German sarcoidosis patients was examined with the Epworth Sleepiness Scale (ESS), the Fatigue Assessment Scale, the Hospital Anxiety and Depression Scale, the Pittsburgh Sleep Quality Index, and the Short-Form Health Survey (SF-8). The patients' ESS mean scores were compared with those obtained from a large general population sample.

**Results:**

Exactly 50% of the patients reached the criterion (ESS > 10) for excessive daytime sleepiness, compared with only 22.1% in the general population. The effect size for the mean score difference between both samples was *d*=0.62. The number of affected organs and the number of concomitant diseases proved to be significant independent predictors of daytime sleepiness. Sleepiness was associated with fatigue (*r*=0.45), anxiety (*r*=0.23), depression (*r*=0.28), sleep problems (*r*=0.23), and detriments in physical (*r*=−0.29) and mental (*r*=−0.28) quality of life.

**Conclusions:**

The issue of excessive daytime sleepiness should be considered in the management of sarcoidosis.

## 1. Introduction

Sarcoidosis is a multisystem inflammatory disease, characterized by epitheloid granulomas [1]. Almost all organs of the body can be involved, but the lungs are the most frequently affected organ. Sarcoidosis patients suffer from a broad spectrum of symptoms such as muscle pain, weight loss, fever, night sweats, cough, breathlessness, and reduced exercise capacity, which results in reduced quality of life (QoL) [2, 3]. Fatigue is a symptom that is particularly prevalent in these patients [4]. This symptom is associated with poor sleep quality and excessive daytime sleepiness (EDS), which is defined as difficulty in maintaining an alert awake state in everyday situations [5]. EDS is associated with insomnia [6], obstructive sleep apnea [7], cognitive impairment [8, 9], cardiovascular diseases [10, 11], psychiatric disorders [12, 13], and low socioeconomic status [14]. The relationship between daytime sleepiness and QoL is less clear. A general population study [15] found small but consistent associations between daytime sleepiness and both physical and mental components of QoL, while among patients suffering from obstructive sleep apnea [16] daytime sleepiness was associated with physical but not mental QoL. Several studies have been performed to analyze fatigue [17, 18] and poor sleep quality [19] in patients suffering from sarcoidosis, but the role daytime sleepiness plays in sarcoidosis patients is largely unknown.

The instrument most often used for measuring daytime sleepiness is the Epworth Sleepiness Scale (ESS) [20]. Convergent, discriminant [21], and ecological validity [22] of the scale have been proved. The items of the ESS were included in the item banks of the Patient-Reported Outcomes Measurement Information System (PROMIS) “sleep disturbance and sleep-related” [23]. Using the ESS, a British study [24] compared sarcoidosis patients with healthy controls and found markedly elevated levels of daytime sleepiness among the sarcoidosis patients (*M* = 8.0 versus *M* = 4.7 in the ESS); however, the sample sizes were low (73 sarcoidosis patients and 82 controls), and the controls were not representative of the general population.

The aims of this study were (a) to determine the degree of EDS in sarcoidosis patients in comparison with the general population and to test psychometric properties of the ESS in a large sample of sarcoidosis patients, (b) to analyze the impact of affected organs and concomitant diseases on EDS, and (c) to investigate associations between daytime sleepiness and QoL.

## 2. Methods

### 2.1. Study Participants

All members of the German Sarcoidosis Society (*n*=4100) were invited to take part in the study and received a booklet with several questionnaires, along with the regular newsletter. Among other scales, the booklet included the ESS. The study was approved by the Ethics Committee of the Medical Faculty of the University of Leipzig. All participating individuals gave informed consent. The inclusion criteria were a diagnosis of sarcoidosis, age 18 years and above, and the willingness to take part in the study.

The scores of the general population were taken from a recent large normative study (*n*=9711) also performed in Germany [25]. We had access to the original data and selected a random subsample (*n*=7990) so that the gender distribution (65.5% women) and the mean age (*M* = 54.2 years) were nearly identical with the sample of the sarcoidosis patients.

### 2.2. Instruments

The ESS [20, 26] was designed to quantify a person's likelihood of falling asleep in several everyday situations and the degree to which sleepiness interferes with their daytime functioning. Eight items have to be answered on a four-point scale. The ESS total score is the sum of the ratings of the items, and it can range from 0 to 24. Scores of 11 and above are frequently considered indicative of EDS [27]. German normative values of the ESS are available [25].

In addition to the ESS, several other questionnaires were used in this study: the Pittsburgh Sleep Quality Index (PSQI) [28], the Fatigue Assessment Scale (FAS) [29], the Hospital Anxiety and Depression Scale (HADS) [30], and the quality of life instrument Short Form Health Survey (SF-8) [31]. Furthermore, self-reported clinical variables (affected organs, concomitant diseases, time since diagnosis, and therapy) were obtained.

### 2.3. Statistical Techniques

Because the proportion of females in the patients' sample was higher than in the normative study, we calculated weighted means for items and sum scores of the general population with the weights corresponding to the gender distribution of the patients' sample to enable unbiased comparisons. The resulting general population means are therefore slightly different from those reported for the total general population study. Group differences in ESS mean scores were statistically tested with *t*-tests. Effect sizes *d* were used to express the mean score difference, related to the pooled standard deviation [32]. A multiple regression analysis (method = enter) was performed to test the combined influence of several predictors of EDS. All calculations were performed with SPSS version 20.

## 3. Results

### 3.1. Sample Characteristics

Of the 4100 patients invited to participate, 1270 (31%) returned the questionnaire. 73 of these questionnaires could not be used because of missing data. In total, data from 1197 participants were ultimately included in the analysis. The sample consisted of 783 females (65.4%) and 414 males with an overall mean age of 54.3 ±11.6 years. The mean time since first diagnosis was 12.8 years (range, 0–64 years). 542 patients (45.3%) were receiving steroid therapy at the time of the survey. The most often affected organs were lungs (89.2%), followed by skin (24.5%) and lymphatic nodes (20.8%). In 39.7% of the patients, only one organ was affected, 28.2% had two affected organs, and 32.1% reported at least three affected organs.

### 3.2. EDS Mean Scores Compared with the General Population

**Figure 1:**
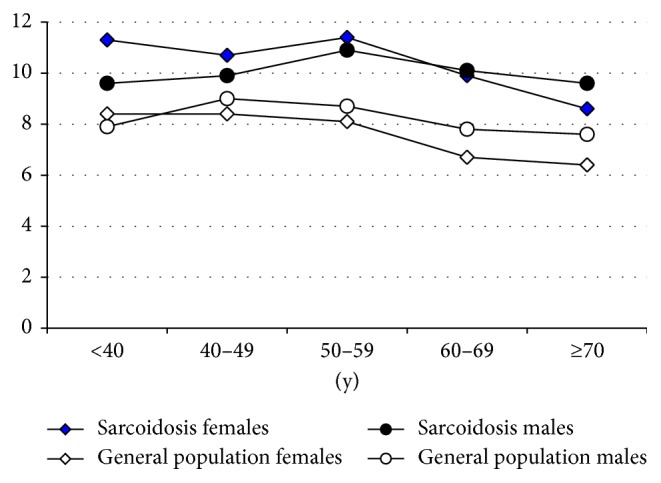
ESS mean scores for sarcoidosis patients and the general population, broken down by gender and age groups.

Figure 1 shows ESS mean scores of the patients and the general population [25]. There was no consistent gender difference between the male and the female patients. In the first three age decades, female patients reported more EDS than male patients, while the trend was opposite for the age groups 60–69 years and ≥70 years.

Every age group of sarcoidosis patients reported higher levels of EDS compared with the general population (Figure 1). The mean of the male patients was 10.18 ± 4.77, while the corresponding mean of the female patients was somewhat higher (10.56 ± 4.91), resulting in a total mean score of 10.43 ±4.87 of the patients. The corresponding mean scores of the general population were 8.26 ± 3.53 (males) and 7.55 ± 3.77 (females). This results in effect sizes of *d* = 0.45 (males) and *d* = 0.71 (females) for the comparison between sarcoidosis patients and the general population; the effect size of the total sample was *d* = 0.62.

Expressed in terms of patients with scores above the cutoff point for excessive daytime sleepiness (ESS > 10), the percentages were 48.1% for the male patients and 51.0% for the female patients. The corresponding percentages from the general population were 25.0% (males) and 20.6% (females) [25]. In the total sample of the sarcoidosis patients, 598 (50.0%) of the 1197 patients had scores above the cutoff point, while the corresponding percentage in the general population was 22.1%.

### 3.3. Item Analyses

Item mean scores are presented in Table 1. As in the general population, the afternoon is the situation with the highest chance to fall asleep. In all eight items, the patients reported more sleepiness than the general population. All items positively contributed to the ESS total score. The reliability (Cronbach's alpha) of the ESS was 0.82.

### 3.4. Effect of Clinical Variables on ESS

Table 2 shows the impact of the affected organs on EDS, expressed in univariate analyses. The sequence of the organs is arranged according to the frequency with which they were affected by the disease. Muscles and bones are the organs with the highest effect sizes. Among the concomitant diseases, sleep apnea was most strongly associated with EDS (*d* = 0.61). There was no statistical significant association between time since diagnosis and daytime sleepiness. Participants receiving steroid therapy were significantly more affected by daytime sleepiness than participants without that therapy.

The results of the multivariate analysis are presented in Table 3. The number of affected organs as well as the number of concomitant diseases proved to be independent factors for predicting EDS. The multiple *r* of this analysis was 0.21.

### 3.5. Associations between Daytime Sleepiness and Other Scales

The correlations between the ESS and other scales are given in Table 4. The strongest association was found for fatigue (*r*=0.44). All the coefficients were statistically significant with *p* < 0.001.

## 4. Discussion

Excessive daytime sleepiness is common among patients suffering from sarcoidosis. Exactly 50% of the sarcoidosis patients fulfilled the criterion of EDS, while only 22.1% of the adult general population reported experiencing EDS. The effect size of the mean score difference was *d* = 0.62. Half a standard deviation (*d* = 0.50) is a frequently used criterion for clinical relevance [33]. The burden of EDS is prevalent in all age groups to a similar degree. Female patients were slightly more affected by EDS than males, though most normative studies [25,34–36] found higher degrees of sleepiness among males.

The ESS proved to be a reliable instrument for measuring EDS in sarcoidosis patients, and the alpha coefficient (0.82) was even higher than the coefficients obtained in other studies [37, 38]. All items contributed positively to the total score, and all items showed a statistically significant difference between the patients and the general population.

If the burden of patients is to be assessed in relation to the burden perceived by healthy controls, it is possible to examine a specifically selected sample of healthy controls or to refer to normative values. We used the second approach, which is more convenient and which provides more profound comparisons. The study with sarcoidosis and idiopathic pulmonary fibrosis patients [24], for example, included a control group of 82 persons with an ESS mean score of 4.7 and a HADS depression mean score of 2.1 which are markedly below the normative scores obtained in larger general population studies. Using such healthy controls can lead to an overestimation of the differences to the patient groups and to an imprecise estimation of the disease burden.

Among the affected organs, muscles and bones were seen to have the highest impact on EDS. A compromised musculoskeletal system may lead to physical inactivity and lack of exercise [39], and a low degree of physical fitness may be associated with EDS. Sarcoidosis patients experience improvements in fatigue levels when they participate in physical exercise programs [40], and they should be encouraged to be physically active even when they are bothered by EDS. However, it should be taken into account that patients whose muscles or bones are affected often have other affected organs too, and that the mean number of concomitant diseases is higher in these patient subgroups.

Concomitant diseases have an impact on EDS, in particular sleep apnea (effect size *d* = 0.61). This corresponds to the findings of other studies [41–43]. Sleep apnea is an interruption of sleep due to the collapse of upper airways, and it can lead to hypoxic episodes. This reduced sleep quality is also associated with daytime sleepiness. The number of affected organs as well as the number of comorbidities proved to be independent prognostic factors for EDS in the multiple regression analysis. This indicates that there are additive effects of the affected organs and concomitant diseases which should be taken into account when treating this patient population. The frequency of several concomitant diseases in the patients' sample was higher than in the general population. Therefore, it is difficult to clarify which proportion of the heightened EDS level is due to sarcoidosis itself and which proportion can be attributed to comorbidity. However, even if the causal relationship between sarcoidosis and comorbidity is unclear, clinicians should know that patients with sarcoidosis experience heightened levels of daytime sleepiness. The clinicians should advise their patients to maintain correct sleep hygiene.

Patients receiving steroid therapy were more strongly affected by EDS than patients without that therapy (Table 2). However, even the patients not receiving steroid therapy had markedly higher daytime sleepiness scores (*M* = 10.1) than people from the general population (*M* = 7.95). This means that sarcoidosis itself (and not the therapy) is the main reason for the heightened levels of sleepiness.

All correlations between the ESS scores and the QoL and mental health scales were statistically significant and clinically meaningful. The strongest association was found with fatigue (*r*=0.45) which underlines the impact of EDS on symptoms such as fatigue. Consistent with the Chinese [15] and the German [25] general population study, daytime sleepiness was associated with both physical and mental components of QoL, and the correlations with the physical component score (*r*=−0.28) and the mental component score (*r*=−0.29) were nearly identical. This might help explain the strong association with fatigue, a concept which also consists of both physical and mental components. This cross-sectional study does not allow for drawing conclusions about causality. EDS might cause fatigue and vice versa.

Some *limitations* of the study should be noted. The presence of sarcoidosis in the sample was not determined by clinical diagnosis, and all clinical data were self-reported. The representativeness of the patients' sample is difficult to assess, thus possibly introducing a selection bias. The univariate analyses presented in Table 2 do not take into account the combined effects of affected organs and concomitant diseases.

In summary, EDS is a problem for one out of every two sarcoidosis patients. Not only sleep quality and depression but also daytime sleepiness should be considered when treating patients suffering from sarcoidosis.

## Figures and Tables

**Table 1:** ESS items and global mean scores.

	Patients		General population		*d*	*p*	*r*_it_	Alpha del.
	M	(SD)	M	(SD)				
1 Sitting/reading	1.43	(1.01)	1.09	(0.89)	0.36	<0.001	0.63	0.78
2 Watching TV	1.97	(0.99)	1.77	(0.93)	0.21	<0.001	0.49	0.81
3 Public place	1.21	(0.97)	0.92	(0.82)	0.32	<0.001	0.66	0.78
4 Car passenger	1.21	(1.05)	0.81	(0.92)	0.41	<0.001	0.56	0.80
5 Afternoon	2.41	(0.85)	2.06	(0.91)	0.40	<0.001	0.36	0.82
6 Sitting/talking to someone	0.39	(0.60)	0.13	(0.40)	0.52	<0.001	0.58	0.80
7 After lunch	1.45	(1.02)	1.01	(0.87)	0.47	<0.001	0.61	0.79
8 Traffic	0.32	(0.62)	0.14	(0.41)	0.35	<0.001	0.48	0.81
ESS total score	10.43	(4.87)	7.95	(3.73)	0.58	<0.001	–	0.82

*Note*. M: mean; SD: standard deviation; d: effect size; *p*: significance; r_it_: part-whole-corrected test-item correlation; alpha del.: Cronbach's alpha if item is deleted.

**Table 2:** The association between clinical variables and daytime sleepiness.

Organs/comorbidity		*N*	M ± SD	*d*	*t*	*p*
*Organs*						
Lungs	Yes	1086	10.5 ± 4.8	0.12	1.08	0.279
	No	111	9.9 ± 5.0			

Skin	Yes	293	10.9 ± 4.9	0.13	1.85	0.065
	No	904	10.3 ± 4.8			

Lymphatic nodes	Yes	249	11.0 ± 4.9	0.14	2.06	0.040
	No	948	10.3 ± 4.9			

Eyes	Yes	191	10.4 ± 5.0	0.00	0.25	0.804
	No	1006	10.4 ± 4.8			

Liver	Yes	141	11.1 ± 5.0	0.16	1.87	0.062
	No	1056	10.3 ± 4.8			

Muscles	Yes	113	12.1 ± 5.1	0.37	3.87	<0.001
	No	1084	10.3 ± 4.8			

Nerves	Yes	108	11.0 ± 5.3	0.12	1.29	0.196
	No	1089	10.4 ± 4.8			

Bones	Yes	105	12.0 ± 5.2	0.34	3.39	0.001
	No	1092	10.3 ± 4.8			

Heart	Yes	95	10.9 ± 4.8	0.10	1.08	0.281
	No	1102	10.4 ± 4.9			

Kidneys	Yes	60	10.7 ± 5.2	0.06	0.49	0.622
	No	1037	10.4 ± 4.8			

Number of organs	<3	813	10.1 ± 4.8	0.23	3.81	<0.001
	≥3	384	11.2 ± 4.9			

*Comorbidity*						
Arterial hypertension^a^	Yes	452	10.6 ± 5.0	0.06	1.02	0.306
	No	738	10.3 ± 4.8			

Disease of thyroid gland^a^	Yes	320	10.6 ± 5.0	0.06	0.99	0.323
	No	869	10.3 ± 4.8			

Obesity (BMI ≥30)^a^	Yes	320	11.3 ± 5.0	0.24	3.87	<0.001
	No	875	10.1 ± 4.8			

Restless legs syndrome^a^	Yes	186	11.4 ± 5.2	0.24	3.00	0.003
	No	998	10.2 ± 4.8			

Diabetes mellitus^a^	Yes	134	10.9 ± 5.2	0.10	1.26	0.209
	No	1060	10.4 ± 4.8			

Sleep apnea^a^	Yes	104	13.1 ± 5.0	0.61	5.95	<0.001
	No	1084	10.1 ± 4.8			

Pulmonary hypertension^a^	Yes	38	10.9 ± 5.5	0.10	0.67	0.503
	No	1138	10.4 ± 4.8			

Number of concomitant diseases	<3	1002	10.1 ± 4.8	0.40	4.30	<0.001
	≥3	195	12.1 ± 5.1			

*Time since diagnosis and therapy*						
Time since diagnosis^a^	≤10 y	625	10.7 ± 4.8	0.10	1.72	0.086
	>10 y	566	10.2 ± 5.0			

Steroid therapy	Yes	542	10.8 ± 4.9	0.14	2.62	0.009
	No	655	10.1 ± 4.8			

*Note*. *d*: effect size; *t*: *t* value of the *t*-test; *p*: significance; ^a^case numbers not added to 1197 due to missing values.

**Table 3:** Demographic and medical factors as predictors of EDS.

	B	SE (B)	Beta	T	*p*
Affected organs (sum)	0.399	0.102	0.116	3.933	<0.001
Comorbidity (sum)	0.545	0.117	0.141	4.652	<0.001
Age	−0.054	0.012	−0.128	4.294	<0.001
Sex (female)	0.030	0.296	0.003	0.101	0.920

**Table 4:** Correlations between the ESS and other scales.

Scale	*r*
PSQI	
Sleep problems	0.23

FAS	
Fatigue	0.44

HADS	
Anxiety	0.23
Depression	0.28

SF-8	
Physical functioning	−0.28
Role-physical	−0.32
Bodily pain	−0.25
General health	−0.25
Vitality	−0.27
Social functioning	−0.30
Role-emotional	−0.32
Mental health	−0.26
Physical component score	−0.29
Mental component score	−0.28

## Data Availability

The data used to support the findings of this study are available from the corresponding author upon request.
